# High-density genetic map construction and QTL mapping of first flower node in pepper (*Capsicum annuum* L.)

**DOI:** 10.1186/s12870-019-1753-7

**Published:** 2019-04-29

**Authors:** Xiao-fen Zhang, Guo-yun Wang, Ting-ting Dong, Bin Chen, He-shan Du, Chang-bao Li, Feng-lan Zhang, Hai-ying Zhang, Yong Xu, Qian Wang, San-sheng Geng

**Affiliations:** 1 Key Laboratory of Biology and Genetic Improvement of Horticultural Crops (North China), Ministry of Agriculture, Beijing Vegetable Research Center, Beijing Academy of Agriculture and Forestry Sciences, Beijing, 100097 People’s Republic of China; 2 College of Horticulture, China Agricultural University, Beijing, 100097 People’s Republic of China

**Keywords:** First flower node, Pepper, High-density genetic map, QTL, Candidate genes

## Abstract

**Background:**

First flower node (FFN) is an important trait for evaluating fruit earliness in pepper (*Capsicum annuum* L.). The trait is controlled by quantitative trait loci (QTL); however, studies have been limited on QTL mapping and genes contributing to the trait.

**Results:**

In this study, we developed a high density genetic map using specific-locus amplified fragment sequencing (SLAF-seq), a high-throughput strategy for de novo single nucleotide polymorphism discovery, based on 146 recombinant inbred lines (RILs) derived from an intraspecific cross between PM702 and FS871. The map contained 9328 SLAF markers on 12 linkage groups (LGs), and spanned a total genetic distance of 2009.69 centimorgan (cM) with an average distance of 0.22 cM. The sequencing depth for the map was 72.39-fold in the male parent, 57.04-fold in the female parent, and 15.65-fold in offspring. Using the genetic map, two major QTLs, named *Ffn2.1* and *Ffn2.2*, identified on LG02 were strongly associated with FFN, with a phenotypic variance explanation of 28.62 and 19.56%, respectively. On the basis of the current annotation of *C. annuum* cv. Criollo de Morelos (CM334), 59 candidate genes were found within the *Ffn2.1* and *Ffn2.2* region, but only 3 of 59 genes were differentially expressed according to the RNA-seq results. Eventually we identified one gene associated with the FFN based on the function through GO, KEGG, and Swiss-prot analysis.

**Conclusions:**

Our research showed that the construction of high-density genetic map using SLAF-seq is a valuable tool for fine QTL mapping. The map we constructed is by far the most saturated complete genetic map of pepper, and using it we conducted fine QTL mapping for the important trait, FFN. QTLs and candidate genes obtained in this study lay a good foundation for the further research on FFN-related genes and other genetic applications in pepper.

**Electronic supplementary material:**

The online version of this article (10.1186/s12870-019-1753-7) contains supplementary material, which is available to authorized users.

## Background

Pepper (*Capsicum annuum* L.) is an economically important vegetable crop worldwide. In pepper, the initiation of the first flower indicates the transition from vegetative to reproductive growth, which is a crucial phase in plant growth, and is regulated by a complex network of flowering-promoter and suppressor genes [1–8]. The first flower node (FFN) on the primary axis is an important criterion for evaluation of maturity in pepper breeding and is linked tightly to flowering time and fruit earliness [9]. Additionally, flowering date, primary axis length, plant height, number of leaves, lateral branch number, and plant width on the primary axis are also linked to fruit earliness [10–14]. In these studies, quantitative trait locis (QTLs) controlling flowering date or flowering earliness were detected on chromosome P02, P04, and P12 [10, 11]; QTLs for primary axis length were identified on chromosome P02 and P09 [10]; QTLs for plant height were detected on chromosome 2, 4, 6, 7 and 8 [14]; QTLs for the lateral branch number were identified on the primary axis on chromosome 2 [14]; and QTLs for the number of leaves were detected on the main stem on all chromosomes except for P09 in pepper [10–13]. The plant height, plant breadth, and maturity are significant and positively correlated with FFN. FFN has been mapped to the chromosomes 2, 3, 5b, 8b and 11 in tomato [15]. In our last report about FFN, we have concluded that the FFN trait is also quantitatively inherited [16]. However, no QTL research on FFN has been conducted in pepper to date.

Molecular markers and genetic maps are important tools for QTL mapping and marker-assisted selection [17–20]. Numerous genetic maps for pepper using either intraspecific or interspecific populations have been constructed based on various marker systems, including restriction fragment-length polymorphism (RFLP) [21, 22], random amplified polymorphic DNA (RAPD) [22, 23], amplified fragment-length polymorphism (AFLP) [22, 24], and simple sequence repeat (SSR) [11, 25]; however, low-density, nonspecificity and incomplete coverage of these simple polymerase chain reaction (PCR)-based molecular markers are limiting factors to the application of these markers in large genomic research for pepper [26, 27]. The advent of next generation sequencing (NGS) technologies has provided an innovative method for genome-wide identification of single nucleotide polymorphism (SNP), insertion–deletion polymorphism, and genotyping of genetic resources [13, 28]. High-density linkage maps are required to study the horticultural traits in pepper along with the development of NGS technology, especially for fine mapping of a locus controlling a specific trait. The availability of large-scale polymorphic markers is a key prerequisite for the construction of a high-density linkage map [14, 29]. In pepper, a set number of high-density linkage maps have been constructed [14, 30–33]. To date, a high-density interspecific BY-SNP map with 5569 SNPs forming 3826 genetic bins represented the highest amount of map saturation [32]. High-density and high-quality genetic maps still are required, especially to illuminate some important traits in pepper.

More recently, a new high-throughput strategy for de novo SNP discovery known as specific-locus amplified fragment sequencing (SLAF-seq) has been developed based on reduced representation genome sequencing and NGS, which is a high-throughput, high-accuracy, rapid and cost-effective strategy for large-scale SNP discovery and genotyping [34]. To date, SLAF-seq has been applied successfully in many species, including soybean [17], wax gourd [29], walnut [35], maize [36], cauliflower [37], spinach [38] and white jute [39]. Furthermore, SLAF-seq combined with bulked segregant analysis also has been applied successfully to pepper [40, 41].

Therefore, the current study applies the SLAF-seq method to construct a high-density linkage map of pepper utilizing the RILs derived from an intraspecific cross between PM702 (*C. annuum*) and FS871 (*C. annuum*). Subsequently, we performed QTL analysis to identify the genomic regions associated with the FFN trait. Finally, we annotated the candidate genes embedded in the major QTLs. The high-density linkage map, QTLs and candidate genes identified by this study will provide useful information for marker-assisted breeding and lay the foundation for the isolation of genes underlying the variation in FFN in pepper.

## Results

### SLAF sequencing and genotyping

According to the pre-restriction enzyme digestion in the reference genome of pepper, we selected the HaeIII restriction enzyme to construct the SLAF library. In total, we generated 106.26 Gb of raw bases and 356.12 Mb of paired-end reads, with each read measuring 100 bp in length (see Additional file 1: Table S1). Among them, 95.14% of the bases were determined to be high quality, with a quality score of at least Q30. The average GC content was 39.42%. We compared rice (*Oryza sativa L. japonica*) as a control with the reference genome to estimate the validity of library construction. We generated 181.80 Mb of total rice bases containing 692,000 paired-end reads with 95.01% Q30 and 45.66% GC content (see Additional file 1:Table S1). The results showed that the rate of paired-end mapped reads was 83.12% in the sample and 92.61% in the control (see Additional file 1: Table S1), and the enzyme digestion efficiency in the control was 92.25% (not shown) with 7.75% of digestion partly in the sequencing reads. These examples show that the paired-end mapped rate for the library construction was normal.

**Fig. 1:**
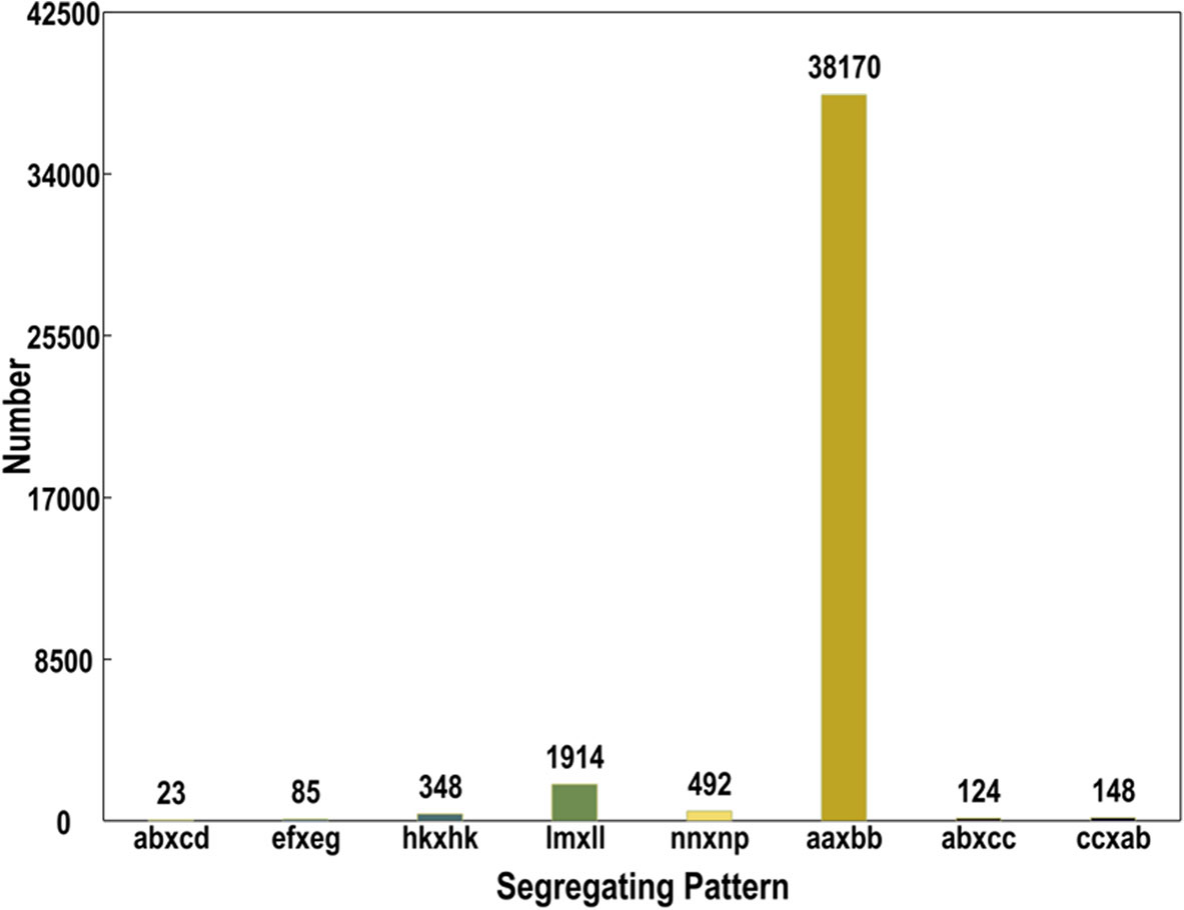
Number of SLAF markers in each segregation patterns

In total, we identified 292,408 SLAFs after all reads were clustered and filtered, and the average sequencing depth of these SLAFs was 30-fold for parents and 9-fold for individual RILs (Table 1). On the basis of the number of SNP mutations and the differences between gene sequences used to analyze the polymorphism of SLAFs, we could identify 70,305 polymorphic SLAFs out of the 292,408 total SLAFs, with a polymorphism rate of 24.04%. After they were filtered, 41,304 SLAFs were successfully encoded and grouped into eight segregation patterns (ab × cd, ef × eg, hk × hk, lm × ll, nn × np, aa × bb, ab × cc, and cc × ab), and only 38,170 SLAFs fell into the aa × bb segregation pattern (Fig. 1). To ensure genotyping quality, we discarded low-quality markers using the five-step filtering process as described in methods, and 9328 SLAF markers with over 50-fold sequence depth for parental lines and over 10-fold sequence depth for offspring individual were qualified for linkage map construction (Table 1; see Additional file 2: Table S2).

**Table 1:** The summary for the number of high-quality SLAFs and SLAF markers on the mapping populations

High-quality SLAFs	
No. SLAFs	292,408
Average depth in male parent	31.75
Average depth in female parent	28.26
Average depth in offspring individual	9.05
Polymorphic SLAFs	
No. of polymorphic SLAFs	70,305
No. of Non-polymorphic SLAFs	221,985
No. of Repetitive SLAF	118
High-quality SLAF markers	
No. of high-quality SLAF markers	9328
Average depth in male parent	72.39
Average depth in female parent	57.04
Average depth in offspring individual	15.65

*No* Indicates number, *SLAF* Specific-locus amplified fragment

### Construction of the genetic map

**Fig. 2:**
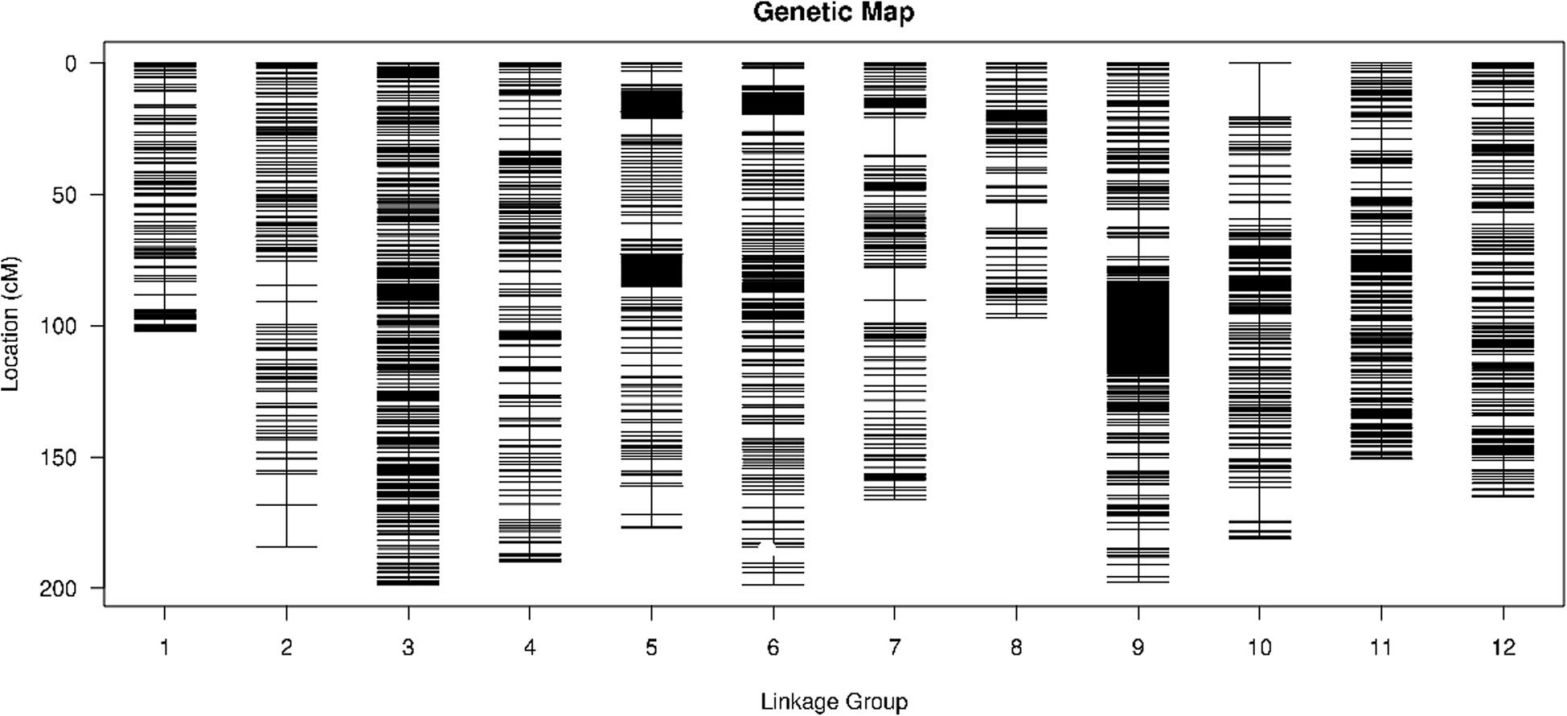
High-density linkage map of pepper. A black bar indicates an SLAF marker. The x-axis and y-axis indicate linkage group number and genetic distance (centimorgan as unit), respectively

We distributed 9328 high-quality SLAF markers into 12 LGs assigned to the corresponding chromosomes in the pepper genome (Fig. 2). The marker names, LGs and genetic position of all markers on the map are included in Additional file 2: Table S2, and the basic characteristics of the genetic map are shown in Table 2 (in the datasets), including the information for markers, genetic distance, gap, and SNPs for each LG. The total genetic distance of the pepper linkage map was 2009.69 centimorgan (cM) long with an average distance of 0.22 cM between adjacent markers. The LG with the greatest number of markers was LG11, which consisted of 1792 markers, with the smallest marker interval (0.08 cM). Conversely, LG08 had the least number of markers (122 markers) with an average density of 0.80 cM. The genetic distances of the 12 LGs ranged from 97.23 cM (LG08) to 198.78 cM (LG06). The largest gap and average gap with a value less than 5 cM, which reflected map uniformity, was 20.56 cM in LG10 and 98.81%, respectively. In addition, we detected a total of 20,775 SNPs ranging from 266 SNPs (LG08) to 4307 SNPs (LG11) on the linkage map with 6531 transversions and 14,244 transitions, for which the number of transitions was 2.18 times that of transversions.

**Table 2:** Basic characteristics of pepper linkage groups

LGID	Total marker	Total distance (cM)	Average distance (cM)	Max gap (cM)	Gap < 5 cM	SNP number	Trv/Tri
LG01	226	102.06	0.45	5.67	98.67%	438	120/318
LG02	347	184.37	0.53	15.90	96.82%	692	202/490
LG03	861	198.61	0.23	2.90	97.33%	1941	624/1317
LG04	455	190.08	0.42	5.75	98.46%	912	284/628
LG05	321	177.12	0.55	10.66	98.75%	709	223/486
LG06	1530	198.78	0.13	6.63	99.54%	2987	949/2038
LG07	641	166.34	0.26	14.38	99.53%	1411	437/974
LG08	122	97.23	0.80	10.07	99.17%	266	86/180
LG09	1381	197.87	0.14	7.68	99.49%	3699	1209/2490
LG10	632	181.35	0.29	20.56	98.57%	1411	419/992
LG11	1792	150.69	0.08	5.75	99.94%	4307	1333/2974
LG12	1020	165.19	0.16	4.80	99.41%	2002	645/1357
Total	9328	2009.69	0.22	20.56	98.81%	20,775	6531/14,244

*LG* Linkage group, *cM* centimorgan, *SNP* Single nucleotide polymorphism, *Trv/Tri* Transversion/transition, *Gap < 5 cM* The average gap between adjacent markers with a value less than 5 cM

### The quality analysis for the high-density genetic map

We constructed a high-density genetic map with a sequencing depth that was 72.39-fold in the male parent, 57.04-fold in the female parent, and 15.65-fold in offspring (Table 1). We evaluated the genetic map using the following indexes. First, we used the integrity of all mapped markers and a haplotype map (see Additional file 3: Figure S3 and Additional file 4: Figure S4) to ensure the accuracy of genotyping. The integrity of all markers on the map among the 146 RILs individuals was 92.80% (on average). In addition, the double crossover percent and missing percent of the linkage map (Table 3) could be reflected in the haplotype maps (see Additional file 4: Figure S4) that were generated for each of the 146 RILs and for the parental controls using 9328 SLAF markers to find genotyping errors [42]. The recombination events of each individual also were displayed in the haplotype maps (see Additional file 4: Figure S4). In this study, the average percent of double crossover, missing and heterozygous fragments was 0.65, 7.54 and 0.30%, respectively, indicating the veracity of the map. Moreover, the RIL populations were well purified and suitable for high-density genetic analysis. The markers allocated on each of the LGs were distributed evenly with a mean interval of 0.22 cM between adjacent markers in spite of the high-frequency of recombination events in the RILs.

**Table 3:** The statistics of evaluation for the pepper linkage map

LGID	Segregation distortion marker	Double crossover percent (%)	Missing percent (%)	Spearman correlation coefficient
LG01	48	1.01	7.12	0.83
LG02	96	0.73	6.97	0.80
LG03	432	0.93	8.22	0.97
LG04	256	0.83	8.20	0.91
LG05	174	0.76	8.52	0.80
LG06	31	0.25	6.11	0.86
LG07	333	0.42	7.64	0.80
LG08	14	0.96	8.08	0.84
LG09	175	0.50	7.95	0.80
LG10	80	0.59	8.40	0.97
LG11	54	0.34	5.88	0.80
LG12	137	0.50	7.41	0.86
Total	1830	0.65	7.54	0.85

*LG* Linkage group

Second, we also used heat maps displaying recombination frequencies between markers on each LG to evaluate the quality of the genetic map by using pair-wise recombination rates for the 9328 SLAF markers (see Additional file 5: Figure S5). These heat maps indicated that the mapped markers were ordered correctly, as the pair-wise recombination rates were considerably low between adjacent markers, and diagonal distribution of the yellow color which indicated the lowest recombination rate generally was shown in the heat map for each LG.

Third, 1830 of the markers showing segregation distortion with *P* < 0.05 were involved in construction of the final map (Table 3), covering 19.62% of all mapped markers, which was considerably lower than that in the interspecific BY-SNP map [32]. The LG with the largest number of markers showing segregation distortion was LG03 with 432, which was followed by LG07 with 333, and the smallest was LG08 with 18.

**Fig. 3:**
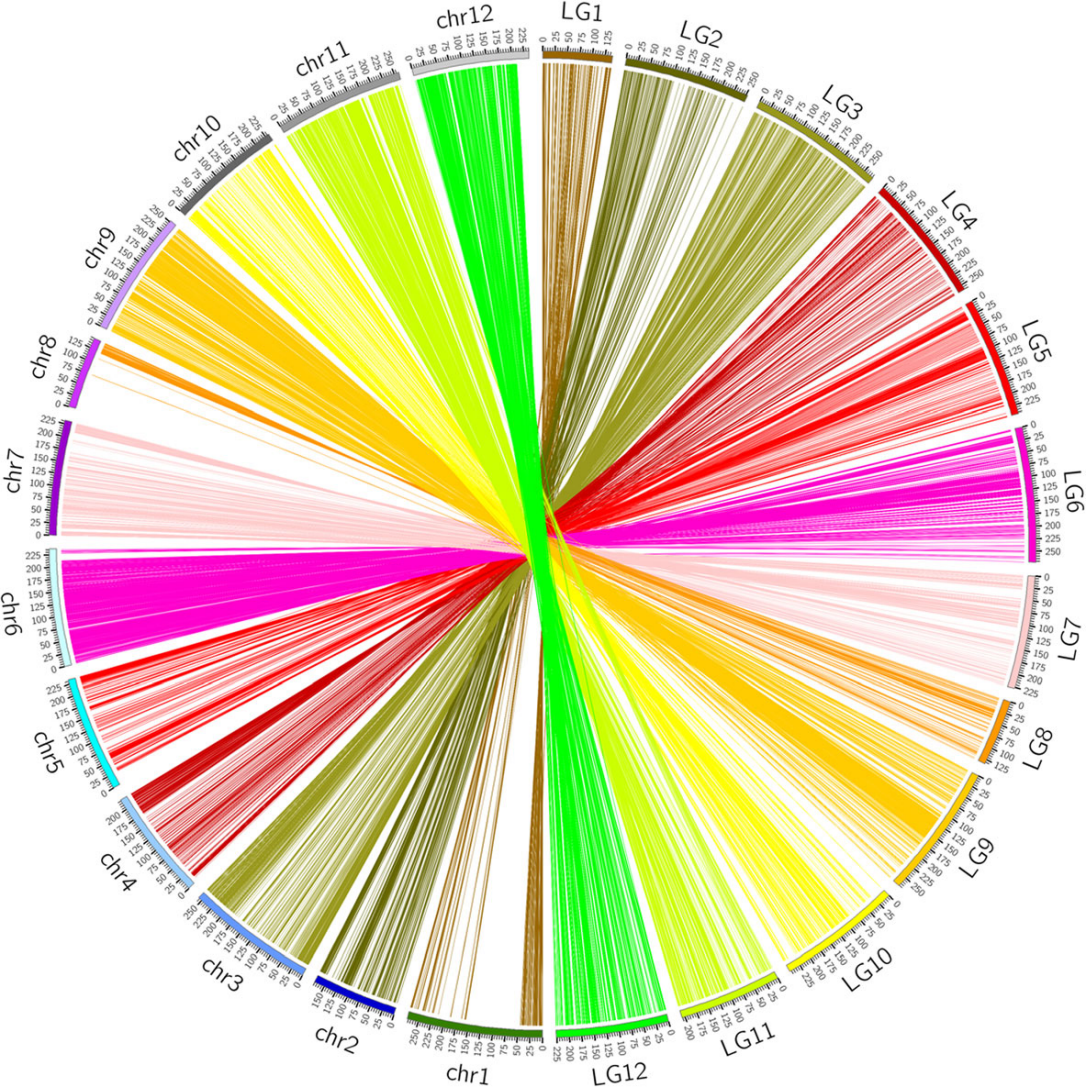
Collinearity between the genetic map and the physical map of pepper. The outer circle indicates the number of chromosomes (ChrID) and linkage groups (LGID); markers located on linkage groups are linked to the corresponding position on chromosomes by different color lines in the inner circle

**Fig. 4:**
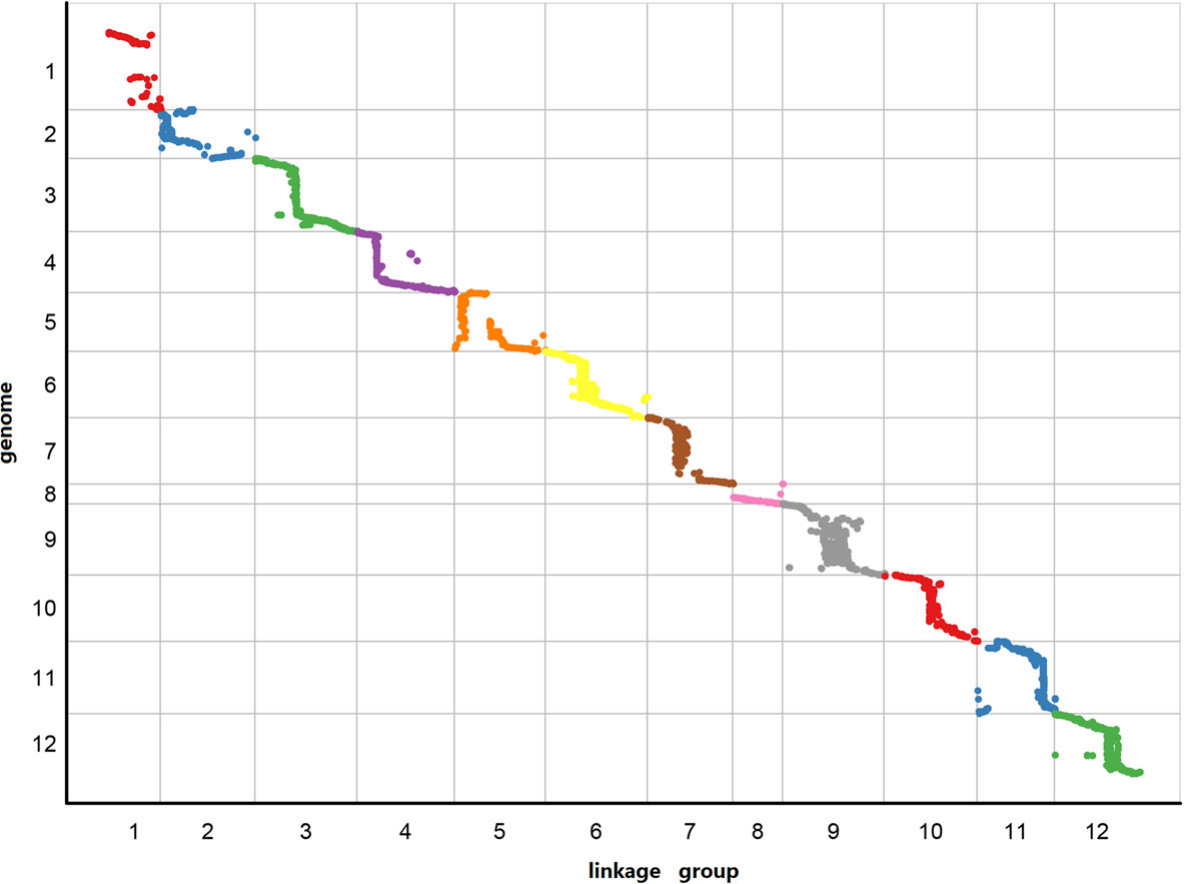
Collinearity scatterplot between the pepper genetic map and the reference genome. The x-axis represents the genetic distance of each linkage group, and the y-axis indicates the physical position in the pepper genome. Different colors represent different linkage groups

Finally, we used the collinearity between the genetic map and the physical map of pepper to evaluate the quality of the genetic map. Utilizing the CM334 reference genome [26], we mapped the 12 LGs and all SLAF markers in the genetic map to the physical map of the pepper genome. The results showed that most of the SLAF markers on the linkage map were in same order as those on the corresponding chromosomes of the physical map even though some inconsistencies in SLAF marker orders were detected in all chromosomes, and 12 LGs were successfully assigned to the 12 corresponding chromosomes (Fig. 3). In addition, Fig. 4 also showed an excellent scatterplot between the physical distances and genetic distances in the 12 LGs. The Spearman correlation coefficient between each LG with a physical map ranged from 0.97 in LG03 and LG10 to 0.80 in LG02, LG05, LG07, LG09 and LG11 (Table 3), and the average Spearman correlation coefficient was 0.85. These results indicated the high accuracy of the linkage map and genotyping.

### QTL mapping for FFN trait in RIL

**Fig. 5:**
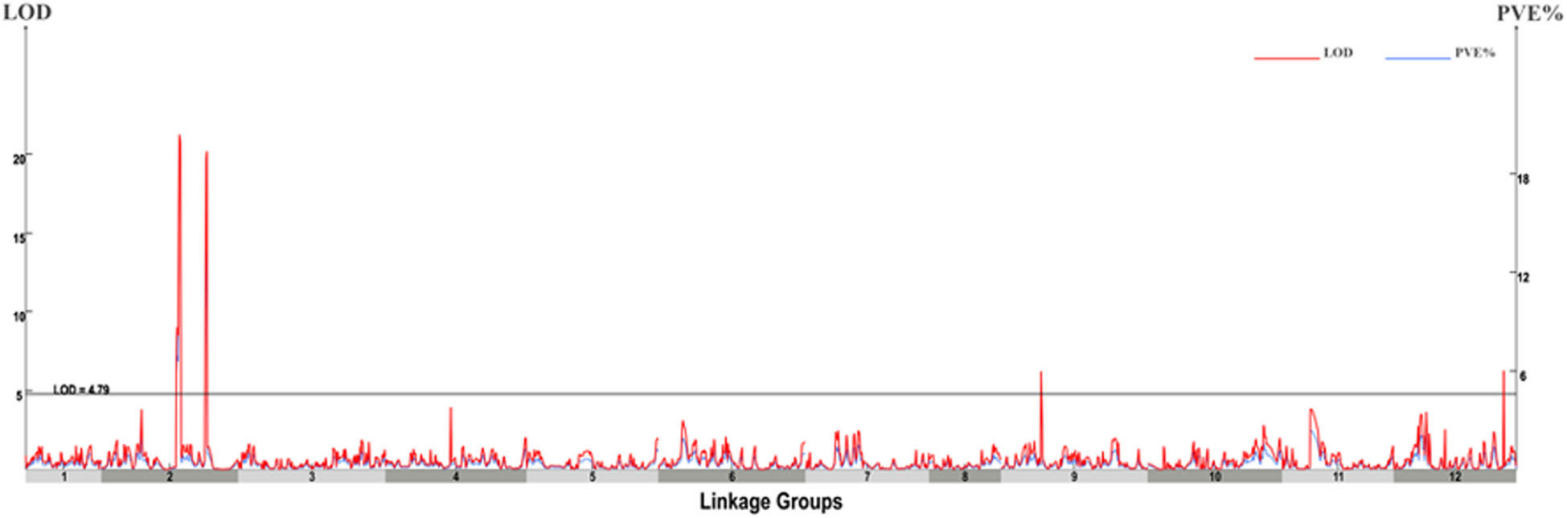
QTL analysis of FFN trait of pepper. The x-axis represents linkage group in pepper and the y-axis indicates LOD values and phenotypic variance explained by additive QTL (PVE%). The gray line indicates LOD threshold of 4.79 (*P* = 0.05)

In term of the high-density genetic map, we performed QTL analysis of FFN loci using the QTL IciMapping V3.3 software [43], and the phenotypic data of FFN of RILs and two parents in three seasons were used (see Additional file 6: Table S6). We set the threshold of LOD scores to evaluate significance (*P* = 0.05) for each marker after 1000 permutations to 4.79, and thus we detected QTLs with LOD scores above 4.79 as effective QTLs. As a result, we identified four QTLs for FFN: *Ffn2.1* and *Ffn2.2* on LG02, and *Ffn9* and *Ffn12* on LG09 and LG12, respectively (Fig. 5; Table 4 in the datasets). *Ffn2.1*, *Ffn2.2* and *Ffn12* harbored two markers separately, whose genetic intervals were 2.25 cM, 1.46 cM and 0.48 cM, correspondingly, whereas *Ffn9* harbored five markers in a genetic distance interval of 53.87–55.29 cM. The peak LOD values for the four QTLs ranged from 6.21 to 21.23 with 5.50 to 28.62% of phenotypic variance explained by an additive QTL. In addition, the additive effects of QTLs varied between − 1.12 and − 0.56 (Table 4). We defined a QTL with a phenotypic variance explanation higher than 15% as a major QTL; otherwise, it was defined as a minor QTL [19]. Therefore, we identified two major QTLs on chromosome 2, *Ffn2.1* and *Ffn2.2*, with a phenotypic variance explanation of 28.62 and 19.56%, respectively, and another two minor QTLs, *Ffn9* and *Ffn12*, on chromosome 9 and chromosome 12 separately. These results suggested that the FFN trait of pepper was regulated primarily by two major QTLs (*Ffn2.1* and *Ffn2.2*) with four markers located at 169.09–169.49 Mb and 162.15–162.61 Mb separately on chromosome 2, which we considered to be the candidate genomic regions.

**Table 4:** QTL analysis for the first flower node in the RILs of pepper

QTL	Chr	Physical distance interval (bp)	Genetic distance interval (cM)	Marker interval	Marker number	LOD	PVE (%)	Additive effect
*Ffn2.1*	2	169,089,654–169,490,486	103.12–105.37	Marker110947–59,204	2	21.23	28.62	−1.11
*Ffn2.2*	2	162,151,445–162,613,220	141.04–142.50	Marker141252–111,037	2	20.17	19.56	−1.12
*Ffn9*	9	51,197,997–96,897,553	53.87–55.29	Marker3157646–3,395,924	5	6.21	6.50	−0.58
*Ffn12*	12	202,951,644–203,633,160	147.77–148.25	Marker1629963–1,686,312	2	6.26	5.50	−0.56

The name of each QTL is defined by the acronym of first flower node trait followed by the number of chromosomes and the position within the chromosome to which the QTL was mapped; *Chr* Chromosome, *LOD* The peak LOD score at which QTL was located, *PVE* Phenotypic variance explained by additive QTL

### Candidate genes and differential expression verification

On the basis of integrating the QTL results, we delimited two loose candidate regions for *Ffn2.1* and *Ffn2.2* into the physical distance of 169.09–169.49 Mb and 162.15–162.61 Mb on chromosome 2, respectively. In total, we predicted that 59 protein coding genes (see Additional file 7: Table S7), including 13 genes without annotation in a public database, would be embed in those regions based on the current annotation of the CM334 reference genome (http://peppergenome.snu.ac.kr/). Among these coding genes, 46, 9 and 42 genes had annotation information in the GO, KEGG and Swiss-Prot databases, respectively. These 59 genes, which participated in many important biological processes, may be related to the FFN trait in pepper and are recommended as important candidate genes for the major QTLs *Ffn2.1* and *Ffn2.2* of pepper for FFN.

**Fig. 6:**
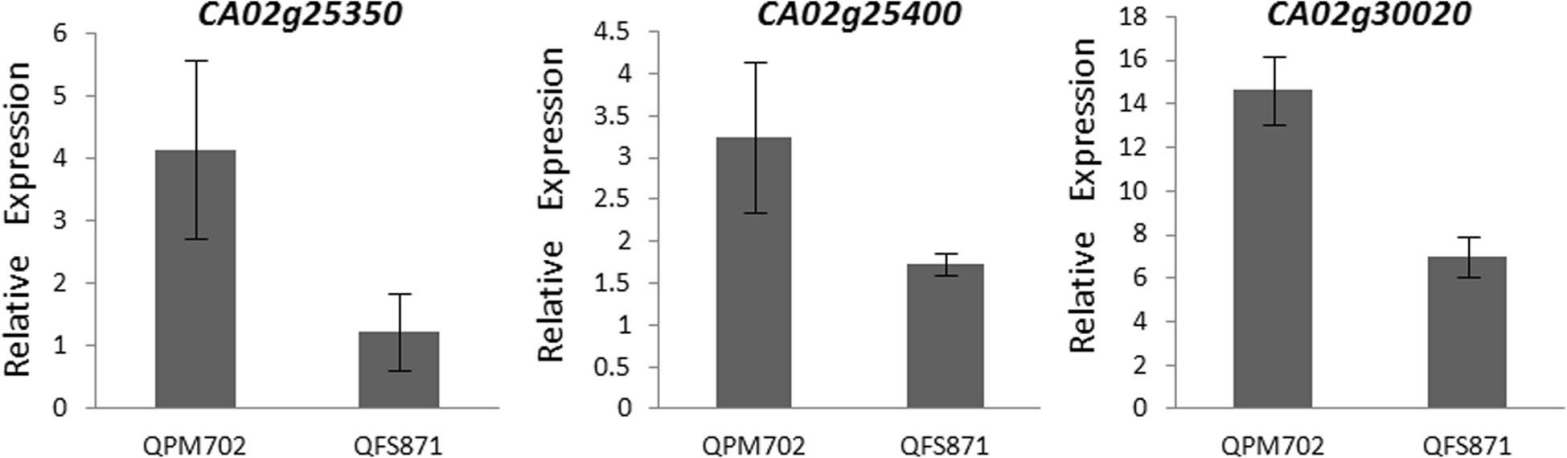
Quantitative expression of three selected genes. Stastical data were obtained from the RNA-seq results of parents QPM702 and QFS871

In order to verify the QTL results and find the target genes, we performed the transcriptome level measurements on the parents. In total, we identified 1774 up-regulated and 2157 down-regulated genes right before the first flower emerging, as well as 2127 up-regulated and 2345 down-regulated genes just after the first flower emerging (see Additional file 8: Table S8). However, only three genes (*CA02g25350*, *CA02g25400* and *CA02g30020*) within the 59 QTL genes were differentially expressed. We also found these three genes were down-regulated in FS871 right before the first flower emerging and the expression level of *CA02g30020* which was annotated as F-box-LRR protein was quite remarkable (Fig. 6) [44].

## Discussion

### Feasibility and advantages of SLAF-seq strategy

In present work, we constructed a high-density genetic map in pepper via the SLAF-seq strategy, which is an effective method for discovery of vast numbers of SNPs and large-scale genotyping [34]. Recently, the SLAF-seq strategy has been used successfully in different species [17, 29, 35–39]; however, this method has rarely been applied in pepper to construct a genetic map for QTL mapping. In contrast to conventional methods of marker development (e.g., RAPD, AFLP and SSR) the SLAF-seq strategy has established several positive characteristics including: high accuracy, high-throughput, shortcuts and cost-effectiveness for large-scale SNP discovery and genotyping [34]. First, we predesigned a scheme that relied on the well-assembled reference genome of pepper to ensure the density, uniformity and efficiency of marker development. For this study, we selected the HaeIII restriction enzyme for the pilot experiment, and fragments ranging from 314 to 394 bp were harvested for sequencing throughout the reference genome to ensure the uniformity of markers. Second, we evaluated the SLAF library by comparing the control genome (*Oryza sativa L. japonica*) with the reference genome to check the reliability and validity of the testing processes, considering the rate of paired-end mapped reads (92.61%), the efficiency of the enzyme (92.25%), and the selection of fragment length (314–394 bp). Third, sequencing depth and sequence quality scores were important to enhance genotyping accuracy. The suggested minimal sequencing depth for each individual was 6-fold when we used the SLAF-seq strategy, and quality scores were not lower than Q30 [34]. We obtained a total of 356.12 Mb paired-end reads in which 95.14% of reads were greater than or equal to Q30, and the sequencing depth was 64.73-fold for parents and 15.66-fold for offspring, which ensured the quantity and quality of markers that we needed to construct a high-quality linkage map. Finally, we selected high-quality SLAF markers based on successive stringent principles. In total, 70,305 of the 292,408 SLAFs were polymorphic with a polymorphism rate of 24.04%, higher than the rate identified by Jiang et al. (2015) [29]. We then filtered the SLAFs using the five-step filtering process to obtain 13,157 high-quality SLAF markers. All of these features demonstrated that the markers had high-throughput, high accuracy, and high efficiency at low cost, suggesting that this SLAF-seq strategy is an effective new tool for large-scale genotyping and markers development and could be used successfully in pepper.

### The high-density genetic map of pepper

We constructed a high-density genetic map using HighMap software after an iterative ordering and error correction strategy, which contained 9328 SLAF markers covering 2009.69 cM on 12 LGs in pepper, with a mean genetic distance of 0.22 cM. Ultimately, we integrated a total of 20,775 SNP markers into the genetic linkage map. To date, our genetic map had high level of saturation, even though it was not saturated sufficiently and had the smallest mean marker distance when compared with the latest version of the pepper genetic map reported by Cheng et al. (2016) [32]. The Cheng et al. (2016) map was an interspecific BY-SNP map using 5569 SNPs forming 3826 genetic bins whose total length was 1628.83 cM with an average bin interval of 0.45 cM [32]. Additionally, we used a 146 individual RIL population in construction of our genetic map, whereas 297 F_2_ individuals were applied in the BY-SNP map. Therefore, our results showed that the high-density genetic map of pepper had a higher density and quality than the previous map. In recent studies, SLAF-seq had been used successfully in RIL [38, 39], F2 [29], BC1 [38] and DH [45] populations in other species. The present study effectively used RIL populations with high recombination frequency to improve the resolution of maps combined with SLAF-seq to construct a high-quality genetic map in pepper.

To evaluate the accuracy of the genetic map, we identified haplotype maps, heat maps, largest gap, average gap with a value less than 5 cM, integrity of map, segregation distortion (*P* < 0.05) rate, and collinearity between the genetic map and the physical map. Results of the haplotype maps and heat maps showed that the high-density SLAF makers in the map were distributed evenly on 12 LGs and were ordered correctly, as the pair-wise recombination rates were considerably lower between adjacent markers. Additionally, the largest gap was smaller than that in the BY map of pepper [13], the average gap value of less than 5 cM was low, and the high integrity of the map reflected the map’s uniformity and veracity. The average Spearman correlation coefficient between the genetic map and the pepper physical map was 0.85, and most SLAF markers were placed accurately within each LG and chromosome as shown in Fig. 4. This suggested high levels of genetic collinearity that strongly indicates the map’s high accuracy and veracity. Assignment of the LGs to corresponding chromosomes indicated the relatively high coverage and confirmed the variation of the *Capsicum* genomes.

We inserted a total of 19.62% segregation distortion markers with *P < 0.05* in the final genetic map, which was considerably lower than that in the interspecific BY-SNP map [32]. Marker segregation distortion may be related to the distant genetic relationship between the parents, as FS871 was cultivated and PM702 was semi-wild [46]; to RIL populations [47]; and to preferential selection or gametic/zygotic selection [48, 49]. The segregation distortion markers inserted in the genetic map could increase the quantity of markers and genomic coverage on the map. If these markers are handled properly, they may be applied to QTL mapping without detrimental effect and could be beneficial for QTL mapping [50, 51].

As shown previously, the various indexes in our high-density genetic map were well balanced and identical to other research findings using the same SLAF-seq strategy [17, 37, 39]. This indicates that our genetic map is accurate, has high throughput, and can be used successfully for QTL mapping. Combined with pepper reference genome sequencing [26], this genetic map can be applied successfully to QTL mapping and candidate gene mining of important traits in pepper.

### QTL mapping and annotated genes for first flower node trait utilizing the high-density genetic map

FFN, as an important criterion to evaluate fruit earliness in pepper, had been mapped on the high-density genetic map. A number of QTLs controlling FFN and its related traits have been identified [1–10, 13–15]. In tomato, QTLs controlling FFN also were located finely at the position [32.5, 37.8] of chromosome 2 [15], but there are no candidate genes present. Moreover, on chromosome 2 in pepper, QTLs controlling plant height, plant width, internode length, flowering date and flowering earliness also were detected [10, 11, 13, 14], which were linked tightly to the FFN [9], suggesting that chromosome 2 is important for evaluation of fruit earliness and pepper breeding. In our study, the two major QTLs, *Ffn2.1* and *Ffn2.2*, explained more than 15% of the phenotypic variation and were located at 169.09–169.49 Mb and 162.15–162.61 Mb on chromosome 2 in the CM334 reference genome, respectively. This location corresponds to their genetic distance intervals of 103.12–105.37 and 141.04–142.50 cM, which were located near *LBN-2.1* and *LBN-2.2* for lateral branch number on the primary axis, and which were detected at 91.4–98.3 and 99.7–104.5 cM on chromosome 2, respectively [14]. *LBN-2.1* and *LBN-2.2* detected in the 120 RILs derived from an intraspecific cross between *C. annuum* Perennial and *C. annuum* Dempsey were located at − 162–165 Mb and − 168–169 Mb on chromosome 2 of CM334 reference genome, respectively [14]. QTLs were similar in physical location between *Ffn2.1*, *Ffn2.2* and *LBN-2.1*, *LBN-2.2* but with a slight difference in position. Although different traits were represented separately by FFN and lateral branch number on the primary axis, they had the same number of changes. So, the QTLs for FFN could be recommended as the same QTLs for the lateral branch number on the primary axis. The different statistical methods, population types and sizes, map saturation or QTL mapping methods may have caused these slight differences. QTLs for the number of leaves on the primary axis were detected at − 143 Mb in the CM334 reference genome on chromosome 2, the low map saturation with an average distance of 5.60 cM and the 147 individual F_2_ population used for QTLs mapping could explain the inconsistency of QTLs [13]. Our genetic map was the highest-density map with the smallest average distance of 0.22 cM using 146 RILs; thus, the QTLs for the FFN should be more accurate than those presented by others.

Because accurate and consistent physical candidate regions could be narrowed down by the reference genome, we can successfully annotated candidate genes combining the whole-genome resequencing data of the parents with highly annotated genomic databases and other bioinformatics methods. Using the EMS mutant materials, the Ilan Paran research team cloned several genes related to the FFN. FASCICULATE, CaJOINTLESS and CaBLIND protein were found to suppress vegetative growth during the reproductive phase in pepper [1, 2, 5]. Whereas *Ca-APETALA2* mapped to chromosome 2 was a flowering repressor gene in pepper [8]. These genes may play a role in controlling the FFN as *AP2* and *CLF* genes, which had been annotated to be related closely to QTLs for the number of leaves on the primary axis of pepper [13]. While in our research results, we found some new genes in the major QTLs (*Ffn2.1*, *Ffn2.2*) related to FFN, which were different from the reported genes mentioned above. According to the CM334 reference genome released in 2014, our QTL and RNA-seq results have annotated 59 and 3931 genes respectively, and 3 genes differentially expressed in parental materials were within 59 genes obtained from QTL results. Therefore, we focused on the expression and function annotation of these three genes, and found that the expression quantity of *CA02g30020* was more pronounced than other two genes. *CA02g25400* was annotated as glycosyltransferase encoding gene and involved in the production of the plant cell wall polysaccharide structural components [52]; while *CA02g25350* was annotated as N-acetyl-Lglutamate synthase gene in the arginine pathway, which finally affected plant stress tolerance [53]. Obviously, these two genes weren’t what we were looking for, only *CA02g30020* annotated as F-box protein and implicated in plant developmental processes might be our target gene [44]. F-box protein is involved in the ubiquitination protein degradation pathway, which is one of the most important biological regulatory systems [54]. Gagne et al. (2002) have identified 694 potential F-box genes in *Arabidopsis thaliana*, making this gene superfamily one of the largest currently known in plants [55]. Also in *Arabidopsis*, He et al. (2017) found a light-induced F-box gene, *FOF2*, was closely related to floral initiation and could promote *FLC* expression to regulate flowering, and the over-expression of *AtFOF2* produced more leaves and delayed flowering, while the mutant *fof2* showed an early flowering phenotype [56]. Our gene expression trend was consistent with the report result, namely that the *CA02g30020* expression in the late flower variety PM702 was significantly higher than that in the early flower variety FS871. So, we hypothesized that *CA02g30020* might also affect the FFN by inhibiting the expression of flower transition gene. We believe that *CA02g30020* is a candidate gene related to the FFN, and the function of the gene remains to be verified by further experiments.

## Conclusions

Our present study for the construction of a high-density genetic map in pepper demonstrates that the SLAF-seq strategy is a powerful method for marker discovery and high-density linkage map construction. Comparative analysis and fine mapping of the FFN trait suggest the high quality and accuracy of this genetic map. On basis of the genetic map, two major QTLs (*Ffn2.1* and *Ffn2.2*) conferring the FFN trait were detected on 0.40 Mb and 0.46 Mb interval on chromosome 2 of pepper for the first time. In addition, we annotated 59 protein coding genes for major QTLs based on the current database of *C. annuum* CM334 and found one key gene combining the RNA-seq results. Hence, the map, QTLs and candidate genes obtained by the present study will be helpful for future basic and applied research with respect to FFN–related traits in pepper.

## Methods

### Plant materials

The node where the first flower developed is taken as the FFN in pepper, and its node number on the primary axis from the node of the cotyledon to the first flower is identified as the characteristic of the FFN trait. PM702 is a semi-wild variety of pepper imported from America National Germplasm Resources Laboratory, belonging to *C. annuum* that produces tall plants with a higher average number of FFN (i.e., 21 nodes). FS871 is a cultivated pepper inbred line, produced by Beijing Vegetable Research Center, Beijing Academy of Agriculture and Forestry Sciences, also belonging to *C. annuum* that produces plants of medium height with a lower average number of FFN (i.e., 10 nodes). A population of 146 F_10_ RIL individuals derived from the intraspecific cross between the two pepper varieties PM702 and FS871 was used for genetic linkage map construction and QTL mapping of FFN. In spring 2015 and spring 2016, plants of the offspring and both parents were grown in the greenhouse at Beijing Vegetable Research Center, Beijing Academy of Agriculture and Forestry Sciences, Beijing, China. And in winter 2015, all plants were grown in open land in Sanya, Hainan, China. Every season, all RIL lines and their parents were planted in a randomzed complete block design with three replicates and at least 6 plants for per line per replicate.

### DNA extraction and SLAF library construction and high-throughput sequencing

We extracted total genomic DNA from young pepper leaves using a plant genomic DNA extraction kit (Tiangen; Beijing, China). An improved variation on SLAF-seq as described by Sun et al. (2013) was used in our experiment [34]. Genomic DNA of both parents and RILs were digested by using the HaeIII restriction enzyme (New England Biolabs; NEB, USA), which was chosen by the reference genome of *C. annuum* cv. Criollo de Morelos 334 (CM334) [26, 57] for pre-restriction enzyme digestion on the basis of the information of the genome size and guanine-cytosine (GC) content. The obtained fragments were combined with a single-nucleotide A using Klenow Fragment (3′ → 5′ exo−, NEB), ligated with dual-index sequencing adaptors [58], and amplified by PCR. The target fragments (314–394 bp in length) were purified, pooled and screened to construct the SLAF library; all as described by Sun et al. (2013) [34]. When the library quality inspection was qualified, the Illumina HiSeq 2500 platform (Illumina, Inc.; San Diego, CA, USA) was applied to sequence the SLAF in the quality-tested library at Biomarker Technologies Corporation in Beijing, China [59]. To verify the reliability and validity of the testing process, we used the genome of *Oryza sativa L. japonica* with a genome size of 38 Mb [60] as a control, and followed the same treatments in accordance with the pepper mapping population.

### Data analysis and development of polymorphic SLAF markers

The dual-index was applied to identify the raw reads obtained by sequencing to find the reads of the samples. The adaptor-filtered reads were evaluated for the sequenced quality score and data size, in which the sequenced quality score was an important indicator to evaluate the error rate of the high-quality single base. We filtered out the reads with quality scores less than Q30 (a quality score of Q30 indicates 0.1% chance of an error, and thus 99.9% confidence). Afterwards, high-quality reads were mapped onto the reference genome of pepper using BWA software 0.7.10 [61], and then the paired-end mapped reads located at the same position with more than 95% identity were grouped into one SLAF locus.

Through a method of comparative genome of the reads, we exploited SLAFs in the parent and offspring to find the polymorphic SLAFs based on SNP mutations. The average sequence depths of SLAFs in parents and offspring were 30-fold and 9-fold, respectively. To ensure the quality of the genetic map, polymorphic SLAFs were filtered by the following five-step filtering process: (1) SLAFs from parents sequencing depth less than 10-fold, (2) SLAFs with more than three SNPs, (3) SLAFs with complete degree below 45%, (4) SLAFs with serious segregation distortion (chi-square test, *P* < 0.001), and (5) SLAFs with redundant tags. These polymorphic SLAFs were classified into eight segregation patterns (ab × cd, ef × eg, hk × hk, lm × ll, nn × np, aa × bb, ab × cc, and cc × ab). As RILs were derived from two fully homozygous parents, only the polymorphic SLAFs with the segregation pattern aa × bb were confirmed as polymorphic SLAF markers.

### High-density genetic linkage map construction and evaluation

We calculated the modified logarithm of odds (MLOD) scores between two markers among all polymorphic SLAF markers [58], and then filtered out markers with MLOD scores less than 8. After that, all high-quality polymorphic SLAF markers were allocated into 12 linkage groups (LGs) and assigned to the corresponding chromosomes. We repeatedly ordered the SLAF markers by MSTmap and corrected genotyping errors or deletions using the SMOOTH algorithm in each LG until all of the markers were mapped appropriately after four or more cycles. We then constructed a high-quality genetic linkage map using HighMap software 1.2.0 [62]. Moreover, genetic distance between adjacent markers was estimated using the Kosambi (1943) mapping function [63]. The linkage map constructed was evaluated based on the integrity of genetic map, haplotype map, heat map, segregation distortion analysis for markers with *P* < 0.05, and collinearity analysis between the genetic map and the physical map of the pepper reference genome. The collinearity between the genetic map and the physical map was conducted using CIRCOS 0.66 software v0.66.

### QTL analysis using high-density genetic linkage map and candidate genes annotation

IciMapping V3.3 software was applied to QTL analysis with multiple environments testing [43], in which additive QTL underlying first flower node of pepper was identified by using the inclusive composite interval mapping (ICIM) method [64]. The threshold of the logarithm of odds (LOD) value was determined by 95% confidence intervals using 1000 permutations to detect significant QTL [65], and each QTL with an LOD above the threshold was detected as significant QTL. We estimated the phenotypic variance explained by individual QTL by the coefficient of determination (*R*^*2*^). In addition, we named individual QTLs by the first letters of first flower node followed by a chromosome number (e.g., *Ffn2*), and a second number indicating the position within the chromosome to which the QTL mapped (e.g., 2.1).

Functional annotation of candidate genes was compared with the nonredundant protein sequences available at Swiss-Prot database using the BLASTX algorithm with default parameters. We then searched the associated hits for their respective gene ontology (GO) terms according to molecular function, biological process, and cellular component ontologies at www.geneontology.org [66]. Finally, the pathways correlated to the candidate gene were detected by Kyoto Encyclopedia of Genes and Genomes (KEGG) analysis.

### RNA-seq analysis

Using a plant genomic RNA extraction kit (Tiangen; Beijing, China), total RNA samples were extracted from the leaves of PM702 and FS871 right before and just after the first flower emerging, with three repetitions and six independent plants per repetition for mixed sampling for each genotype. The RNA sequencing (RNA-seq) library was prepared according to Xiang et al. (2011), and sequencing was performed on an IIIumina HiSequation 2000 (Frasergen, Wuhan, China) [67]. Raw reads were filtered to remove adaptor sequences, low quality tags (tags with unknown nucleotides *N* > 10%), and reads with more than 50% low quality (≤5) bases. To identify genes that were differentially expressed between PM702 and FS871, the gene expression levels were quantified in terms of FPKM (fragments per kilobase of exon per million mapped fragments) using DEseq2 with default parameters. The DEGs were identified based on the following thresholds: absolute of log2 (fold-change) > 1 (<− 1) and q-value (false discovery rate (FDR)) < 0.05. Finally, all DEGs were mapped to terms in the GO, KEGG and Swiss-prot databases, ready for the comparison.

## Additional files

Additional file 1: **Table S1.** Consequence of the comparison control dates and sample dates with reference genome. (DOC 31 kb)

Additional file 2: **Table S2.** List of marker names, linkage groups, and genetic distances of 9328 SLAF markers in the genetic map of pepper. (XLS 1118 kb)

Additional file 3: **Figure S3.** The integrity figure of all mapped markers in all individuals. The x-axis and the y-axis represent the 146 recombinant inbred lines and their integrities, respectively. (PNG 68 kb)

Additional file 4: **Figure S4.** Haplotype maps of the pepper genetic map. Each row represents a marker, and each chromosome of each individual is shown in the column. Green indicates female parent, and correspondingly, blue indicates the male parent, and red indicates heterozygosity. The color change in the same column represents a recombination event. LG indicates the linkage group. (PNG 1589 kb)

Additional file 5: **Figure S5.** Heat maps for the linkage relationship between markers for each linkage group. Each cell represents the recombination rate between markers. Yellow, red, and purple indicates the minimum, median, and maximum recombination rate, respectively. LG indicates the linkage group. (PNG 1849 kb)

Additional file 6: **Table S6.** Phenotypic data of FFN of RILs and parents in three seasons. (XLSX 16 kb)

Additional file 7: **Table S7.** List of 59 candidate genes located in the major QTLs *Ffn2.1* and *Ffn2.2*. (XLSX 21 kb)

Additional file 8: **Table S8.** Summary of differential expression genes just before and after the first flower emerging. (XLSX 1683 kb)

## Abbreviations

AFLP: Amplified fragment-length polymorphism
cM: Centimorgan
CM334: *C. annuum* cv. Criollo de Morelos 334
FFN: First flower node
GC: Guanine-cytosine
GO: Gene ontology
ICIM: Inclusive composite interval mapping
KEGG: Kyoto Encyclopedia of Genes and Genomes
LG: Linkage group
LOD: Logarithm of odds
MLOD: Modified logarithm of odds
NGS: Next generation sequencing
PCR: Polymerase chain reaction
QTL: Quantitative trait loci
RAPD: Random amplified polymorphic DNA
RFLP: Restriction fragment-length polymorphism
RILs: Recombination inbred lines
RNA-seq: RNA-sequencing
SLAF: Specific-length amplified fragments
SLAF-seq: Specific-length amplified fragments sequencing
SNP: Single-nucleotide polymorphism
SSR: Simple sequence repeat
