# Supplementary material for: High-density genetic map construction and QTL mapping of first flower node in pepper (Capsicum annuum L.)
Source: BMC Plant Biol. 2019 Apr 29;19:167. doi: 10.1186/s12870-019-1753-7 (PMC6489210; doi:10.1186/s12870-019-1753-7)
Supplement: Supplementary file 1 — Table S1. Consequence of the comparison control dates and sample dates with reference genome. (DOC 31 kb) [file 12870_2019_1753_MOESM1_ESM.doc]

**Table S1. Consequence of the comparison control dates and sample dates with reference genome**

| Items | Sample (pepper) | Control (rice) |
| --- | --- | --- |
| Total bases | 106,260,111,298 | 181,803,022 |
| Total reads | 356,120,132 | 692,000 |
| Q30 percentage (%) | 95.14 | 95.01 |
| GC percentage (%) | 39.42 | 45.66 |
| Paired-end mapped reads (%) | 83.12 | 92.61 |
| Single-end mapped reads (%) | 2.65 | 2.49 |
| Unmapped reads (%) | 14.23 | 4.90 |

Q30 indicates a 1% chance of an error and thus 99% confidence. GC represents guanine-cytosine.
