# Supplementary figures and images for: High-density genetic map construction and QTL mapping of first flower node in pepper (Capsicum annuum L.)
Source: BMC Plant Biol. 2019 Apr 29;19:167. doi: 10.1186/s12870-019-1753-7 (PMC6489210; doi:10.1186/s12870-019-1753-7)

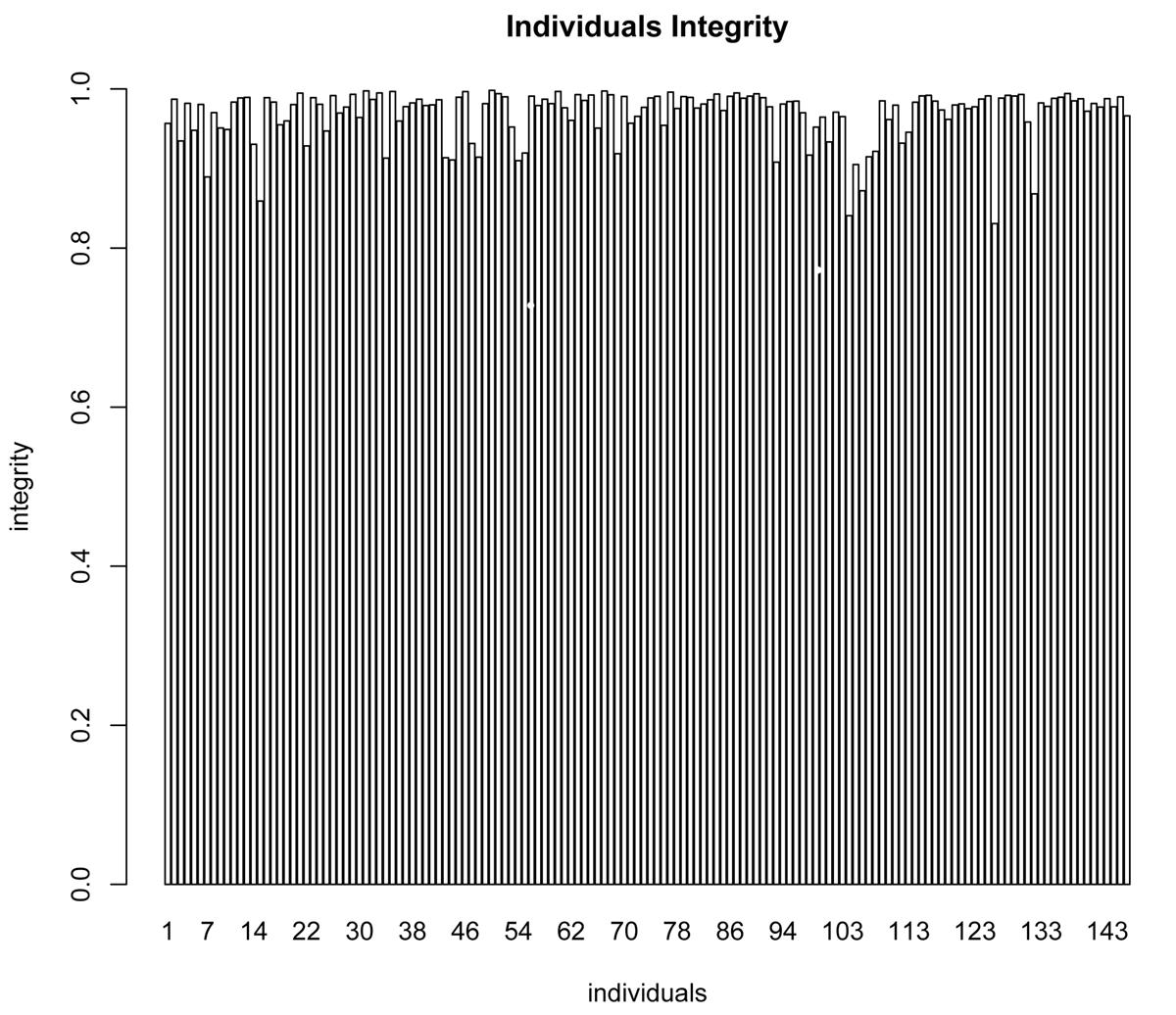

Supplement: Supplementary file 3 — Figure S3. The integrity figure of all mapped markers in all individuals. The x-axis and the y-axis represent the 146 recombinant inbred lines and their integrities, respectively. (PNG 68 kb) [file 12870_2019_1753_MOESM3_ESM.png]

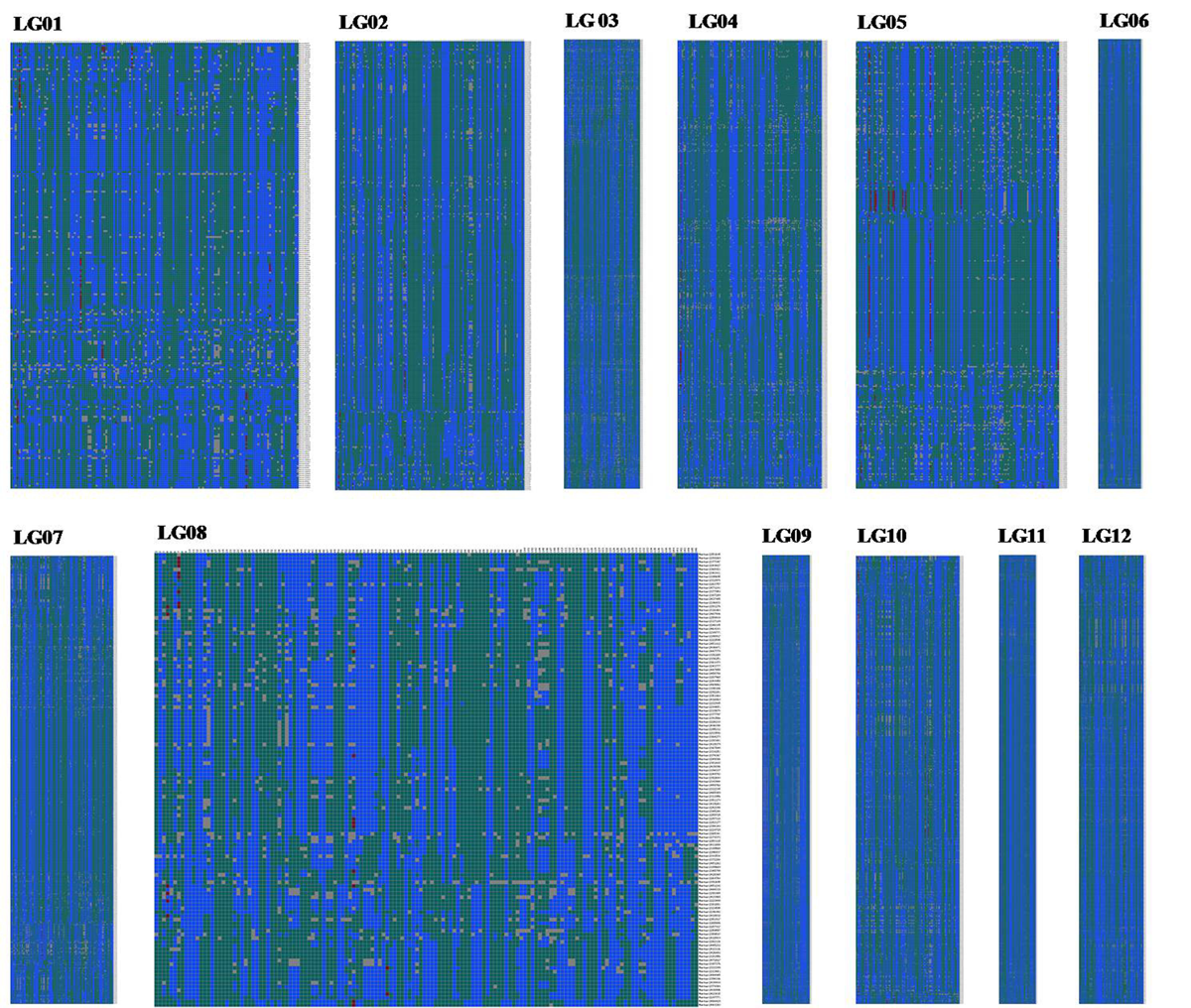

Supplement: Supplementary file 4 — Figure S4. Haplotype maps of the pepper genetic map. Each row represents a marker, and each chromosome of each individual is shown in the column. Green indicates female parent, and correspondingly, blue indicates the male parent, and red indicates heterozygosity. The color change in the same column represents a recombination event. LG indicates the linkage group. (PNG 1589 kb) [file 12870_2019_1753_MOESM4_ESM.png]

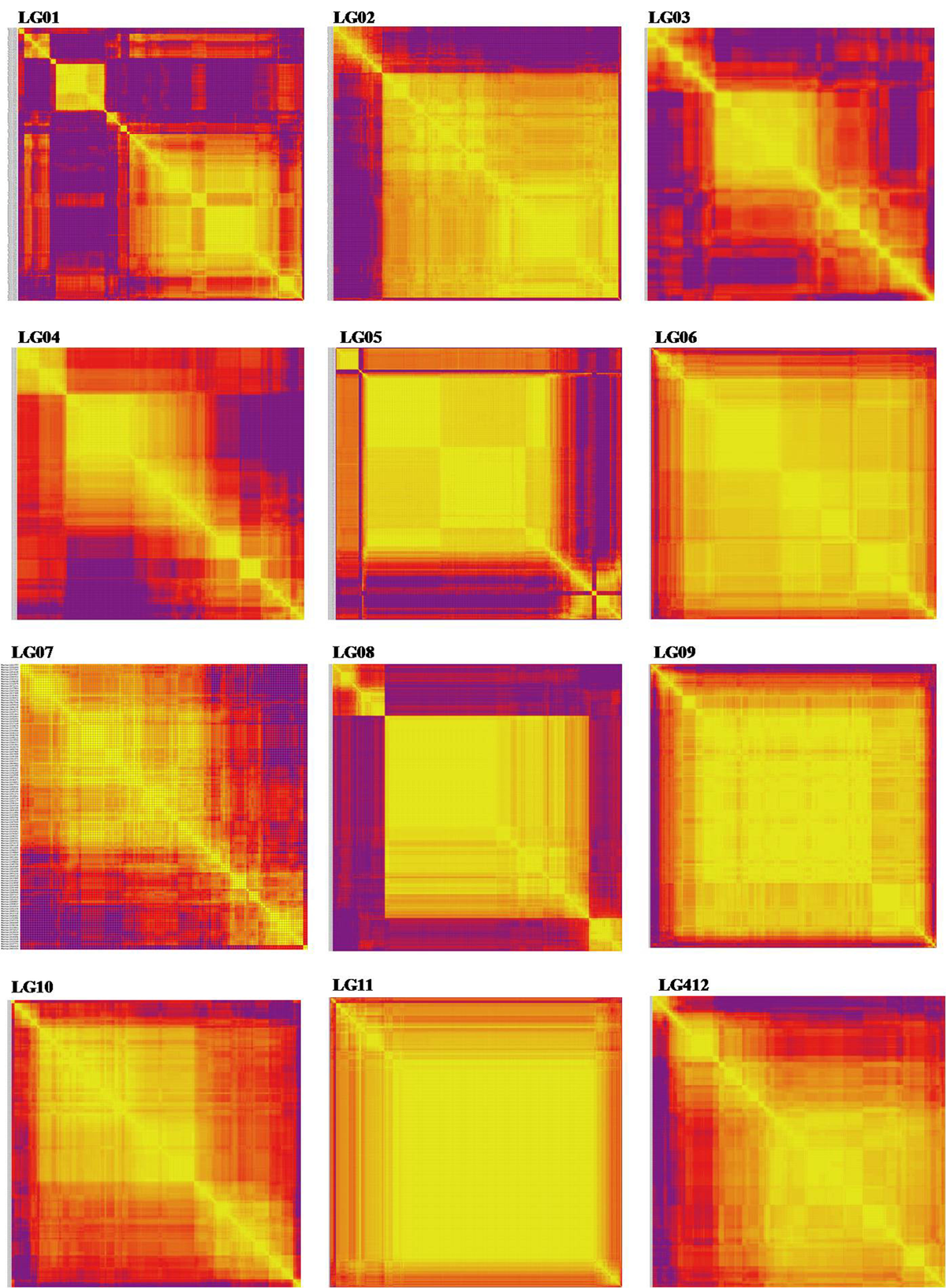

Supplement: Supplementary file 5 — Figure S5. Heat maps for the linkage relationship between markers for each linkage group. Each cell represents the recombination rate between markers. Yellow, red, and purple indicates the minimum, median, and maximum recombination rate, respectively. LG indicates the linkage group. (PNG 1849 kb) [file 12870_2019_1753_MOESM5_ESM.png]
